# A Novel Self-Expanding Transcatheter Mitral Valve with Dual Annulus/Valve Diameter

**DOI:** 10.3390/jfb16070250

**Published:** 2025-07-07

**Authors:** Irina Yu. Zhuravleva

**Affiliations:** E. Meshalkin National Medical Research Center, Ministry of Health of Russian Federation, 15 Rechkunovskaya St., Novosibirsk 630055, Russia; juravl_irina@mail.ru

**Keywords:** nitinol, self-expanding stent, transcatheter mitral valve, TMVR

## Abstract

**Background:** The development of transcatheter mitral valves (TMVs) represents a major advancement in cardiology, driven in part by the growing elderly population. Elderly patients frequently suffer from secondary mitral regurgitation but are often ineligible for surgical valve replacement due to high procedural risks. This study aimed to develop a self-expanding TMV stent fabricated from a single nitinol tube, featuring two distinct central zones: a smaller-diameter valve-containing segment and a larger-diameter anchoring segment for the mitral annulus. **Methods:** We used the COMSOL Multiphysics 6.0 software package for biotechnical engineering. Prototypes of stents and valves were manufactured in five sizes following a 22 Fr delivery system compatibility assessment and pulsatile-flow testing. **Results:** We bioengineered a novel stent design with an integrated porcine pericardial valve. The stents were laser-cut from nitinol tubes (4.5 mm outer diameter, 0.45 mm wall thickness) and heat-treated to achieve spatial configurations compatible with fibrous ring diameters of 40, 42, 44, 46, and 48 mm. Pericardial leaflets and coverings were then mounted onto the stents. The resulting valves were successfully loaded into a 24 Fr delivery system and exhibited proper opening and closing function under pulsatile-flow testing. **Conclusions:** Our findings confirm the feasibility of a single-component, dual-diameter TMV stent, offering a promising solution for high-risk patients with mitral regurgitation.

## 1. Introduction

Degenerative valvular heart diseases (VHDs) are increasingly prevalent in developed countries due to population aging. While only 1% of individuals aged 45–54 are affected, the incidence rises sharply to 8–10% in those over 75, with secondary mitral regurgitation (MR) being the most common lesion [1,2,3]. Severe MR often necessitates surgical intervention [4,5,6,7]; however, open-heart surgery carries substantial risks in elderly patients. Those over 75 face a threefold higher rate of major complications and a fourfold increase in perioperative mortality compared to patients under 50, with mortality reaching 17% in octogenarians [8,9,10].

A breakthrough for high-risk elderly patients with degenerative aortic stenosis came in the early 21st century with transcatheter aortic valve implantation (TAVI), pioneered by A. Cribier in 2002 [9]. As evidence of its safety and efficacy accumulated, TAVI expanded to include intermediate- and even low-risk patients [11]. Today, surgeons and interventional cardiologists have access to approximately 10 TAVI systems, most featuring self-expanding nitinol stents. Unlike balloon-expandable designs, these valves employ multiple anchoring zones and adaptable geometries, accommodating anatomical variations in the aortic root [12].

**Figure 1:**
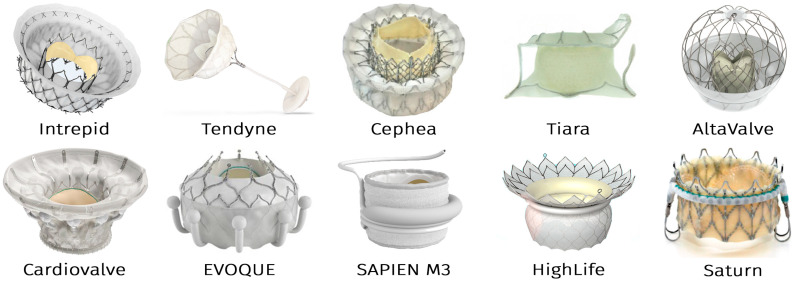
Design of transcatheter mitral valves in clinical trials.

The transcatheter mitral valve (TMV) era began in 2012 with the first-in-human implantation of the CardiAQ™ valve by Lars Søndergaard (Copenhagen, Denmark) [13]. After a decade of development, 10 TMV systems are now in clinical trials (Figure 1):

- Intrepid (Medtronic)
- Tendyne and Cephea (Abbott Vascular)
- Tiara (NeoVasc)
- AltaValve (4C Medical)
- Cardiovalve (Cardiovalve Ltd.)
- EVOQUE (formerly CardiAQ) and SAPIEN M3 (Edwards Lifesciences)
- HighLife (HighLife Medical)
- Saturn (InnovHeart)

Notably, nine of these devices utilize self-expanding nitinol stents, with the balloon-expandable SAPIEN M3 being the sole exception [14,15,16].

The slower progress of transcatheter mitral valve (TMV) development compared to transcatheter aortic valves (TAVR) arises from the mitral valve’s complex anatomy and dynamic functional demands. Unlike the aortic valve, the mitral valve is a multicomponent structure composed of a highly mobile D-shaped fibrous annulus that undergoes significant conformational changes during the cardiac cycle and two leaflets anchored by chordae tendineae to the left ventricular papillary muscles. Dysfunction of any component can compromise valve performance, complicating the design of effective TMVs.

**Figure 2:**
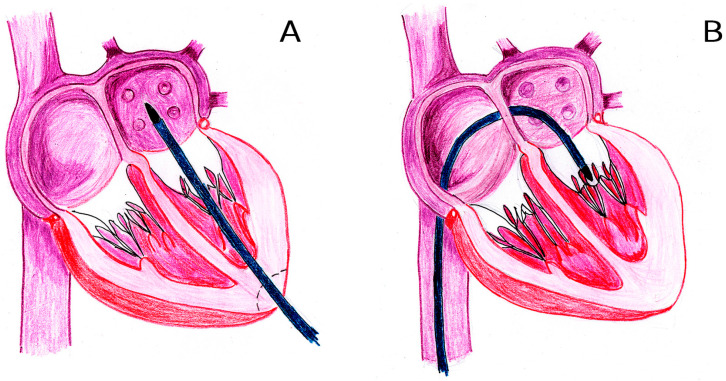
Mitral valve transcatheter approaches: transapical (**A**) and endovenous transseptal (**B**).

A key challenge is the pathological dilation of the mitral annulus in secondary mitral regurgitation (MR), when annular diameters often exceed 45 mm (compared to the normal range of 26–30 mm). To prevent left ventricular overload post-implantation, TMVs must accommodate this dilation while minimizing prosthetic valve diameter. This has driven the development of dual-stent designs, such as those used in the Intrepid, Tendyne, Cephea, and Cardiovalve systems [13]. However, these stents—comprising two interconnected components—cannot be crimped into low-profile delivery systems (DS). Consequently, devices like the Intrepid and Tendyne require high-profile DSs (34 Fr and 35 Fr, respectively), restricting their use to transapical access (Figure 2A).

Even TMVs designed for transseptal access (Figure 2B) face limitations, with most DS diameters exceeding 28 Fr (over 9 mm) [14]. This presents a critical challenge for endovenous delivery, given the anatomical constraints of the common femoral vein (8–11 mm) and the great saphenous vein (6–11 mm).

The choice between transapical and transseptal access remains contentious [17,18,19,20]. Although the transseptal approach is favored for its minimally invasive nature, it demands DS miniaturization. Current DS designs often generate substantial atrial septal defects, necessitating adjunctive occluder implantation in many cases [21].

Leveraging nitinol’s unique properties—particularly its capacity to achieve complex 3D geometries through stepwise thermal shaping—enables the fabrication of single-component stents with a dual-diameter central zone: an outer profile for anchoring in the mitral annulus and an inner profile containing the valve. A critical design consideration is the initial diameter of the nitinol tube, which directly determines crimping potential and, consequently, the delivery system (DS) profile. This approach relies exclusively on radial force for anchoring, presenting both advantages and limitations.

Current self-expanding TMV systems employ five to six primary anchoring strategies besides radial forces. Often, these strategies are used in combination (Figure S1) [17,21]:

1. Apical tethering with an epicardial pad;
2. Atrial winglets or subannular piercing hooks;
3. Stent elements grasping native mitral leaflets;
4. Atrial and ventricular segments clamping leaflets/annulus;
5. Two-component systems with external docking elements;
6. Internal anchoring along the left atrial circumference (AltaValve).

A defining feature of most contemporary transcatheter mitral valves (TMVs) is the incorporation of an atrial inflow cuff, evident in designs such as the Intrepid, Tendyne, Cephea, Tiara, Cardiovalve, and HighLife systems (Figure 1). This cuff fulfills two critical roles: preventing ventricular migration of the prosthesis and mitigating paraprosthetic leaks.

The anatomically complex target zone, further complicated by pathological changes in mitral regurgitation, has driven the development of diverse engineering solutions. This diversity underscores the absence of a universal optimal TMV design, with each approach balancing trade-offs among deliverability, anchoring stability, and hemodynamic performance.

Aim of this work: To develop a novel self-expanding transcatheter mitral valve (TMV) stent with the following key innovations:

- Single-component stent laser-cut from a monolithic segment of the NiTi tube;
- Dual-diameter central zone: a valve containing a smaller-diameter segment and a larger-diameter segment anchoring within the mitral annulus.
- This design aims to balance the competing demands of:
- Low-profile crimping (enabling transseptal delivery via reduced delivery system dimensions);
- Secure annular anchoring (ensuring prosthesis stability under dynamic cardiac forces).

By integrating these features into a single-component architecture, the proposed TMV seeks to circumvent the limitations of multi-part systems while optimizing deliverability and functional performance.

## 2. Materials and Methods

The material chosen for the stents was a superelastic nitinol tube according to the ASTM F2063-18; standard [22] (Baoji Hanz Metal Material Co., Ltd., Shaanxi, China). The leaflets and the stent covering elements were made of porcine pericardium with a thickness of 0.2–0.25 mm, preserved with 5% ethylene glycol diglycidyl ether (DE) solution. Porcine pericardium was sourced from local slaughterhouses.

### 2.1. Stent Design Requirements

At the first stage, based on known data on the mitral valve anatomy and functioning, the following design criteria were established:

- The bioprosthesis can be implanted using the transapical or endovascular transseptal approach; therefore, it must be packaged in a delivery system with an external diameter of no more than 24 Fr.
- To prevent bioprosthetic dislocation to both the left ventricle and left atrium, its stent should have 3 reliable anchoring zones: in the patient’s mitral valve annulus, in the LV, and in the LA.
- In order to reduce the cross-sectional area of the valve, the stent must have two concentric elements in the mitral annulus area. The outer one, of larger diameter, fixing in the mitral valve annulus, must have elastic deformability to match annular dynamics during the cardiac cycle. The inner concentric element must carry the valve itself and maintain valve circularity with a diameter of 28–30 mm, regardless of the deformations of the mitral annulus.
- The design of the bioprosthesis as a whole should not provoke the left ventricle outflow tract (LVOT) obstruction, regardless of the anatomy of the patient’s left heart; the laser-cut template must be unified but adaptable to annular sizes (40–48 mm) with minimal post-shaping deviations of critical dimensional parameters for all stent sizes.

### 2.2. Stent Engineering and Manufacturing

Biotechnical engineering of the stents was carried out using the COMSOL Multiphysics 6.0 software package (COMSOL Inc., Stockholm, Sweden), choosing as a basic reference the range of the most common mitral annulus diameters in secondary mitral regurgitation: 40–48 mm. It was taken into account that in order to create radial forces fixing the stent in the annulus, it is necessary that the outer diameter of the stent exceed the diameter of the annulus by at least 3 mm.

Laser cutting of a nitinol tube with an outer diameter of 4.5 mm and wall thickness of 0.45 mm was carried out using a *dwg file. Laser cutting was performed at the «Polytechnic» Science and Technology Park of the Belarusian National Technical University (Minsk, Belarus) on the StarCut Tube femtosecond laser machine (Rofin, Hamburg, Germany) (Figure S2).

The stent shaping was performed by heat treatment on titanium 3D printed matrices (Logeeks Ltd., Novosibirsk, Russia). The stents were then post-processed using sandblasting and electrochemical polishing.

### 2.3. Biomaterial Mounting

To manufacture the valves, we used porcine pericardium preserved with DE (N.Vorozhtsov Novosibirsk Institute of Organic Chemistry SB RAS, Novosibirsk, Russia) using the method described previously in detail [23]. Under aseptic conditions (ISO Class 5), thickness measurement and precision cutting of biomaterial were performed using specially developed templates with a laser machine (Figure S3) “MELAS-Cardio” (Institute of Laser Physics SB RAS, Novosibirsk, Russia). For cutting the valvular details, pericardium areas with a different thickness were selected: 0.21–0.25 mm for leaflets, and 0.18–0.20 mm for atrial cuff covering. The cut details were fixed to the stent with suture monofilament material PTFE 7/0 0 (Ecoflone, St-Peterburg, Russia) using surgical instruments and techniques.

### 2.4. Crimping and Loading into the Delivery System

To validate the transcatheter delivery of the valve, a loading test was performed using the 22 Fr transapical DS (Figure S4), previously developed at the «Polytechnic» Science and Technology Park of the Belarusian National Technical University (Minsk, Belarus). Manual compression (without dedicated equipment) was carried out to simulate worst-case conditions.

### 2.5. Pulsatile-Flow Testing

Valve performance was assessed using a MedEng hydrodynamics tester (Russia; Figure S5) compliant with ISO 5840-3:2021 [24] (Annex C). This unit included a sample fixation device, pressure sensors DMP 331 (BD Sensors, Russia; accuracy: 0.5% span), a digital pressure control unit, an oil-free air compressor, a 10 L water tank, and a fluid collection device.

Each tested sample was hermetically fixed with customized rubber gaskets in the testing box. The forward flow pressure was 20 ± 1 mm Hg; back flow pressure was 120 ± 5 mm Hg, peak differential pressure across the closed valve was 100 mm Hg, the cycle rate was 70 bpm, and simulated cardiac outputs were 3 L/min and 7 L/min. The valve functioning was documented by triple-camera video recording (inflow/outflow/axial views (Videos S1–S3)).

## 3. Results

Based on the design requirements outlined in Section 2.1, the key dimensions of the valve were established (Table 1).

The sizing strategy integrated the following physiological and engineering considerations. Given the proportional enlargement of left heart structures in mitral regurgitation, the valve diameters were scaled accordingly: a 28 mm valved segment for annuli measuring 40–44 mm, and a 30 mm valved segment for annuli measuring 46–48 mm. To ensure adequate radial fixation forces, the outer diameter of the central anchoring zone was designed to exceed the native annular diameter by 4–5 mm (Figure S6).

### 3.1. Implementation of Design Parameters

**Figure 3:**
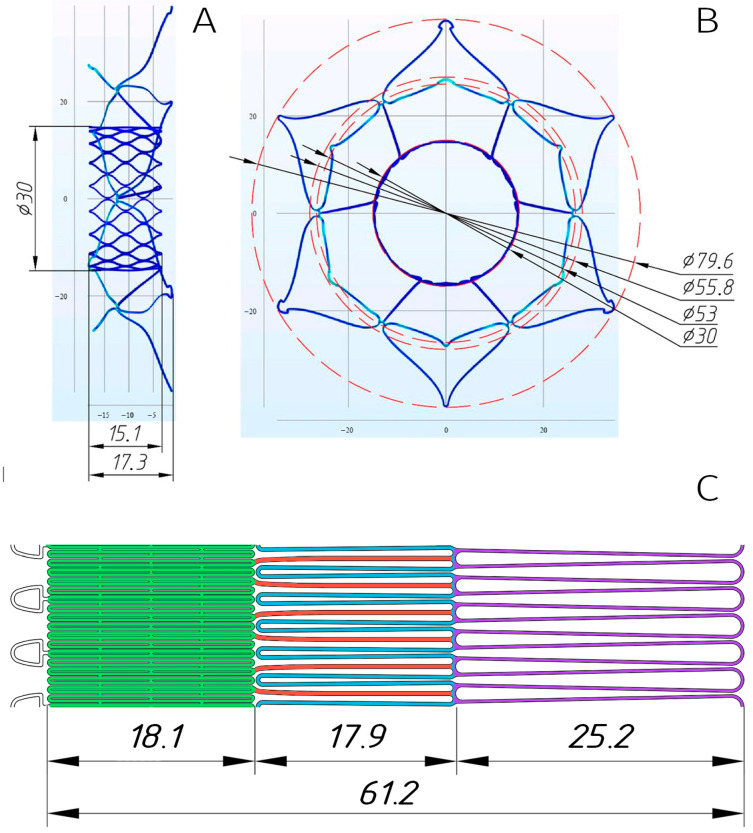
Three-dimensional model of the stent designed for implantation in a 48 mm mitral annulus (**A**,**B**). Planar laser-cutting template with color-coded functional elements (**C**): valve-containing segment (green), ventricular anchors (blue), structural joining elements (red), and atrial “cuff” (lilac).

In accordance with the defined parameters, five distinct stent models were developed to accommodate the target diameter ranges (Figure 3A,B and Figure S7). These models incorporated 4–5 mm oversizing of anchoring zones to the mitral annulus, dual-diameter (valve/annulus) architecture, and height optimization (≤20 mm total).

Following verification of the stents’ spatial configurations, laser-cutting drawings (.dwg files) were generated from nitinol tubes (see Section 2.2), with the planar template illustrated in Figure 3C.

Notably, this work diverged from conventional approaches used in aortic valve development, which typically rely on population-averaged anatomical data [25,26]. Unlike the aortic root—where structural dimensions remain relatively uniform in aortic stenosis—the left heart’s anatomical parameters, particularly their proportional relationships in mitral regurgitation (MR), exhibit significant individual variability. Consequently, our objective was to design a mitral valve prosthesis adaptable to diverse left ventricular (LV) and left atrial (LA) geometries.

### 3.2. Valve Modeling and Biomaterial Optimization

The subsequent stage involved 3D valve modeling to develop laser-cutting templates for porcine pericardium, to optimize suture lines for biomaterial fixation, and to verify the valve’s spatial configuration.

**Figure 4:**
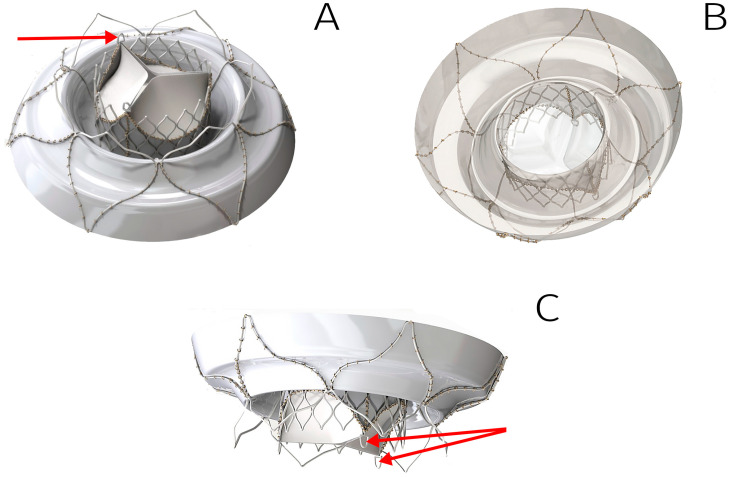
Three-dimensional model of the TMV developed: outflow view (**A**), inflow view (**B**), side view (**C**). Arrows indicate the fixing elements to anchor inside DS.

Results demonstrated robust biomaterial fixation on the stent, with no risk of deformation or excessive suture-line stress during valve straightening into a tubular shape for delivery system loading (Figure 4). Notably, we deemed it unnecessary to tissue-cover the ventricular portion of the stent, as this reduces the risk of left ventricular outflow tract (LVOT) obstruction and facilitates crimping of the stent’s central segment.

### 3.3. Implementation of TMV Prototypes

**Figure 5:**
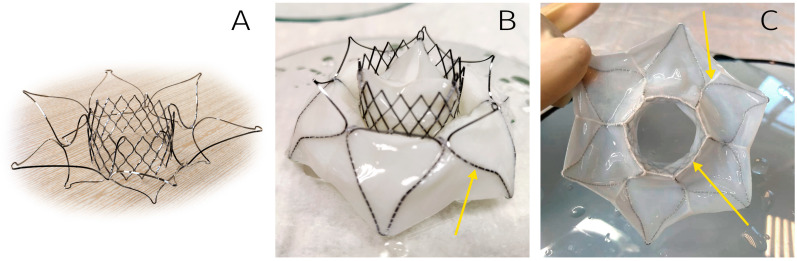
The TMV developed: bare stent (**A**), outflow view (**B**), and inflow view (**C**). Arrows indicate suture lines.

Following meticulous optimization of stent and valve designs, five TMV prototypes were fabricated. The workflow included sequential steps:

1. Laser cutting of nitinol tubes;
2. Thermal shaping and post-processing of stents;
3. Biomaterial fixation onto stents (Figure 5).

### 3.4. Delivery System Compatibility Assessment

Next, the feasibility of crimping and loading the prosthesis into a low-profile delivery system (DS) was evaluated. This test highlights the critical relationship between prosthesis diameter and deliverability, particularly relevant for transcatheter mitral interventions where anatomical variability is substantial.

**Figure 6:**
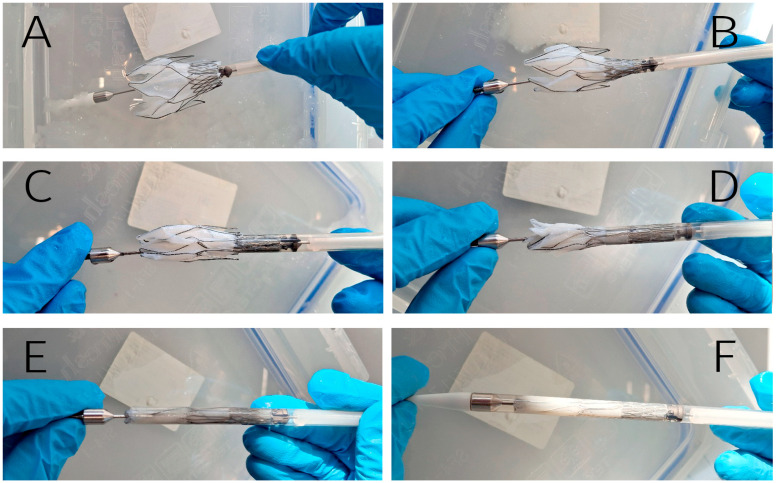
Crimping and loading into the 22 Fr delivery system. The stent’s fixing elements are anchored inside the DS (**A**); sequential loading of valve-containing segment (**B**,**C**); the middle part (joining elements and outer ring) is placed in the delivery system (**D**); complete crimped configuration (**E**); final prepared DS ready for implantation (**F**).

Valves sized for 40–44 mm annuli were successfully crimped and loaded into a 22 Fr DS without complications. However, the largest valve (designed for 48 mm annuli) exhibited challenges during crimping, with excessive tightness observed within the catheter lumen (Figure 6). As a result of this, increased friction during deployment, biomaterial damage, and/or stent frame deformation may occur.

### 3.5. Hemodynamic Performance Evaluation

The study concluded with a comprehensive hydrodynamic assessment using a pulse duplicator system to evaluate: leaflet kinematics during systolic closure and diastolic opening, and also coaptation integrity under physiological pressures (120/80 mmHg).

**Figure 7:**
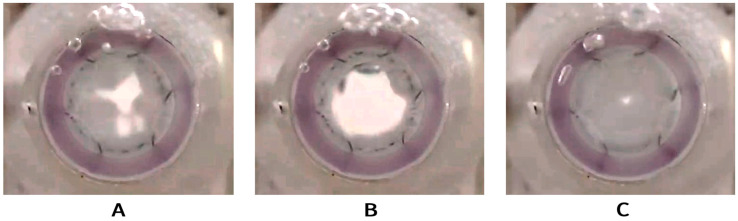
Opening and closing function of the developed valve being tested in pulse duplicator; inflow view. Semi-open valve (**A**), fully open valve (**B**), and fully closed valve (**C**).

The valve-containing segment, when compressed by the outer anchoring zone in the gasket, maintained a circularity with no observed impediments to leaflet function (Figure 7, Videos S1–S3).

This experiment enabled qualitative validation of the valve’s functional performance while identifying potential areas for iterative design improvement.

## 4. Discussion

The clinical performance and long-term outcomes of transcatheter mitral valves (TMVs) are critically dependent on device design. While preliminary data from single-center studies (typically involving 1–3 devices and small patient cohorts) offer initial insights, robust comparative evidence remains limited. This gap is anticipated to diminish with 22 active clinical trials currently underway [27], whose findings will critically inform future transcatheter mitral valve implantation (TMVI) technologies.

Clinical assessment of TMVs in the perioperative period focuses on some paramount endpoints: technical success of the implantation procedure and freedom from early complications, including bleeding, prosthesis migration, LVOT obstruction, paravalvular leaks, and embolization of vessels or cardiac chambers by a non-deployed/insufficiently deployed device [16,27,28,29]. Each of these criteria is associated with several anatomical factors and the adequacy of engineering solutions in the development of TMVs and their DS.

These outcomes are intrinsically linked to patient-specific anatomical factors (e.g., annular dynamics, LV dimensions); device engineering solutions (stent architecture, anchoring mechanisms); and delivery system refinements (profile, deployment control).

Technical success depends, in particular, on the diameter of the delivery system [27,28]: the smaller its diameter, the lower the risk of vessel/DS mismatch during endovenous transseptal implantation. During transapical implantation, a decrease in the DS diameter will help reduce the risk of intra- and postoperative bleeding. We hypothesize that single-step implantation (e.g., Intrepid, Tendyne) offers higher technical success rates compared to multistep systems (e.g., Sapien M3, HighLife, Saturn), which require sequential implantation of docking elements and valve components [17,21,27]. Thus, the engineering task is to reduce the diameter of the DS, and the engineering challenge is to create a valve design that can be placed in such a system. To address these challenges, we chose to cut a tube with an outer diameter of 4.5 mm (approximately 14 Fr). With an outer diameter of 22 Fr (7.3 mm) and a wall thickness of 0.4 mm, the sheath has an inner diameter of 6.5 mm (more than 19 Fr). The stent design is such that it can be stretched and crimped to the diameter of the original tube when cooled. The remaining lumen space (~2 mm) was allocated for biomaterial when packing the valve in DS. It turned out that this is sufficient for 40–44 mm valves. However, the largest valve (48 mm) exhibited significant crimping and loading challenges, which can result in significant sheath retraction resistance or even DS component failure, and also valve structural compromise and irreversible leaflet damage [30].

Paravalvular leaks arise from the D-shaped and saddle-shaped anatomy of the mitral annulus, posing unique sealing challenges. While valves like the Tiara and Tendyne address this through asymmetrical D-shaped stents [31], most transcatheter mitral valves (TMVs) rely on stent cuffs lined with biological or synthetic tissue to mitigate leaks [16]. In dual-component systems, where the valve-containing segment is smaller than the stent’s outer perimeter, the biomaterial must also cover the transition zone to ensure sealing [32].

The cuff not only prevents the paraprosthetic regurgitation, the risks of which are always high when implanting a circular valve into a non-circular mitral annulus, but also prevents the migration of the prosthesis towards the LV, especially after its endothelialization in the late postoperative period. Therefore, the wider the cuff, the higher its importance in preventing leaks and dislocations. At the same time, excessive cuff width can result in at least two problems. First, the nitinol cuff edges risk traumatizing the atrial wall. Despite left atrial (LA) dilation in mitral regurgitation (MR), individual anatomical variability means oversized cuffs may exceed LA dimensions. Second, the stent cuff must be covered with biomaterial to prevent leaks, and the wider the cuff ring, the more material must be packed into the delivery system, which leads to difficulties in crimping and an increase in the diameter of the delivery system. Taking into account both of these reasons, we considered the cuff of the valve we developed to be excessive. Future design iterations will prioritize cuff reduction to balance sealing efficacy with procedural safety and DS compatibility.

The LVOT obstruction problem is influenced by anatomical variability, including the aorto–mitral annular angle, displacement of the anterior mitral leaflet toward the LVOT, or severe mitral annular calcification [27,33]. These factors currently restrict transcatheter mitral valve implantation (TMVI) eligibility in many patients. The problem with the anterior leaflet can be solved by laceration procedures [29], and the problem with a smaller aorto–mitral annular angle can be solved by setting a low profile, as in the Intrepid (17–18 mm high) and Cephea [31] valves. That is reasonable, and the device we develop has a 15 mm high valve-containing part (Figure 4). However, using the LV as an additional anchoring zone is a very tempting idea. It is limited by the common opinion that all nitinol stent elements contacting blood must be isolated with a biomaterial to prevent thrombus formation and the connective tissue growth on the bare nitinol. We offer other approaches aimed at improving hemo- and cytocompatibility of the stent surface. Thus, we demonstrated earlier a significant increase in the compatibility of the nitinol surface with endothelial cells as a result of magnetron sputtering of titanium oxynitride [34,35]. Other nano- or micro-coatings can be used for these purposes. Uncovered ventricular fixators will not be significantly obstructive to the blood outflow from the LV, which will allow for optimizing neo-LVOT in a large cohort of patients, and, accordingly, expand the indications for TMVI. As for our device, this method makes it possible to lengthen the ventricular fixators in order to enhance valve anchoring stability in the LV and prevent its migration into the left atrium. We intend to do this when improving our TMV.

Embolization of cardiac chambers or vessels by non-deployed or partially deployed devices is a rare but critical complication. For a self-expanding nitinol valve, it depends not on the design, but on the ability of the delivery system to retain it during deployment, as well as on the deployment speed upon DS release. In turn, It is limited by the common opinion that all nitinol stent elements contacting blood must be isolated with a biomaterial to prevent the thrombus formation and the connective tissue growth on the bare nitinol. the deployment kinetics are influenced by the austenite finish temperature (Af) achieved during thermal shaping, underscoring the need for strict control of Af during stent manufacturing. These problems are quite solvable during the development and validation of the device, and the target Af temperature should be strictly controlled during the stent manufacture.

Retrievability during or after deployment is also one of the main advantages of the valve design, as it allows for more precise positioning. However, among current systems, only Tendyne is completely recapturable at all stages of implantation [31]. The other valves are only partially retrievable. Our valve allows the valve-containing portion to be retained in the delivery system until the atrial cuff and ventricular clamps are fully deployed, and at this stage, it allows for repositioning. However, its full manipulation properties can only be fully assessed in upcoming studies on large animals.

As already mentioned, the clinical experience with TMVI is still very limited. For a systematic review published in 2022 [31], only 12 studies were recruited that presented the results of at least a 30-day follow-up. In 2023, the 2-year results of a multicenter study (31 clinics) were published, obtained on a cohort of 400 patients with various implanted valves [36]. In 2023–2025, a number of more clinical studies appeared [37,38,39,40,41,42]. Summarizing the data obtained from these studies, the following conclusions can be drawn:

- The Tendyne valve is the most often used by surgeons and interventional cardiologists. There are two reasons for this. Firstly, this valve was the first to receive the CE Mark. Secondly, it seems more reliable to surgeons due to its additional anchoring mechanism: apical tethering with an epicardial pad.
- Technical success of the implantation procedure is quite high and amounts to 95–100%, and this indicator depends to a lesser extent on the valve model and to a greater extent on the experience of the clinic. Such high indicators indicate good engineering of the entire “valve + delivery device” system.
- Despite the fact that all dedicated transcatheter valves correct MR well, 1-year cardiovascular mortality remains high: 17–20%. According to Ludwig et al. [36], 2-year all-cause mortality is 38%, with similar values for Tendyne and other studied valves.

In the coming years, TMVs will be improved as they are the only chance for life for high-risk patients, and TMVI is more cost-effective compared to surgical mitral valve implantation [43].

## 5. Study Limitations

The primary limitation of this study lies in its focus on the initial conceptual prototype of the self-expanding TMV rather than a finalized device. While our objective was to validate an engineering solution for a single-component, dual-diameter valve, minor design refinements—specifically adjustments to cuff dimensions and ventricular fixator geometry—remain necessary. Although the opening and closing function of the developed valve has been roughly estimated, further tests in a pulse duplicator are required to obtain the quantitative characteristics, using a statistically significant number of valves of each size, as well as various flow characteristics (frequency, opening and closing pressures, “cardiac output”). In addition, it will be necessary to evaluate valve functionality and biocompatibility under physiologic conditions in large-animal models to assess long-term performance and safety. All these steps are critical to advancing the design toward clinical applicability.

## 6. Conclusions

1. Our findings confirm the feasibility of a single-component, dual-diameter TMV stent, offering a promising solution for high-risk patients with mitral regurgitation.
2. Biological material can be fixed to these stents with no risk of deformation or excessive suture-line stress during valve straightening into a tubular shape for 22 Fr delivery system loading.
3. The developed valves have a dimensional geometry suitable for implantation in the mitral position and demonstrate adequate opening/closing function in the pulse duplicator.
4. Before the next iteration of testing, further stent architecture optimization is required:
  - Lengthening of ventricular fixation elements, improving intra-LV anchoring;
  - Reduction in the cuff width to facilitate crimping of valves > 44 mm.

## Figures and Tables

**Table 1:** Dimensional specifications of the developed transcatheter mitral valve.

**Parameters**	**Size, mm**				
Recipient’s mitral annulus = outer diameter of implanted device	40–41	42–43	44–45	46–47	48–49
Real outer diameter of central anchoring zone	45.0	47.0	49.0	51.0	53.0
Valve diameter	28.0	28.0	28.0	30.0	30.0
Valve stent height	15.6	15.6	15.6	15.1	15.1
Total height, no more than	20.0				

## Data Availability

The data presented in this study are available on request from the corresponding author, due to the privacy agreement between the authors and the E. Meshalkin National Research Center.

## Supplementary Materials

The following supporting information can be downloaded at: https://www.mdpi.com/article/10.3390/jfb16070250/s1, Figure S1: Transcatheter mitral valve anchoring mechanisms; Figure S2: StarCut Tube femtosecond laser machine; Figure S3: The biomaterial laser cutting machine “MELAS-Cardio”; Figure S4: Transcatheter 22 Fr delivery system developed at the «Polytechnic» Science and Technology Park of the Belarusian National Technical University (Minsk, Belarus); Figure S5: Pulsatile-flow tester; Figure S6: 3D models of stents for implantation in the patients’ annuli with diameters of 40 mm (A), 42 mm (B), 44 mm (C), and 46 mm (D). Video S1: Pulsatile-flow testing; inflow view; Video S2: Pulsatile-flow testing; axial view; Video S3: Pulsatile-flow testing; outflow view.

## Abbreviations

The following abbreviations are used in this manuscript:

DS	Delivery system
LA	Left atrium
LV	Left ventricle
LVOT	Left ventricle outflow tract
MR	Mitral regurgitation
NiTi	Nitinol
TAV	Transcatheter aortic valve
TAVI	Transcatheter aortic valve implantation
TMV	Transcatheter mitral valve
TMVI	Transcatheter mitral valve implantation
VHD	Valvular heart disease
