# Supplementary material for: A Novel Self-Expanding Transcatheter Mitral Valve with Dual Annulus/Valve Diameter
Source: J Funct Biomater. 2025 Jul 7;16(7):250. doi: 10.3390/jfb16070250 (PMC12295116; doi:10.3390/jfb16070250)
Supplement: Supplementary file 1 [file jfb-16-00250-s001.zip › suppl_figures.pdf]

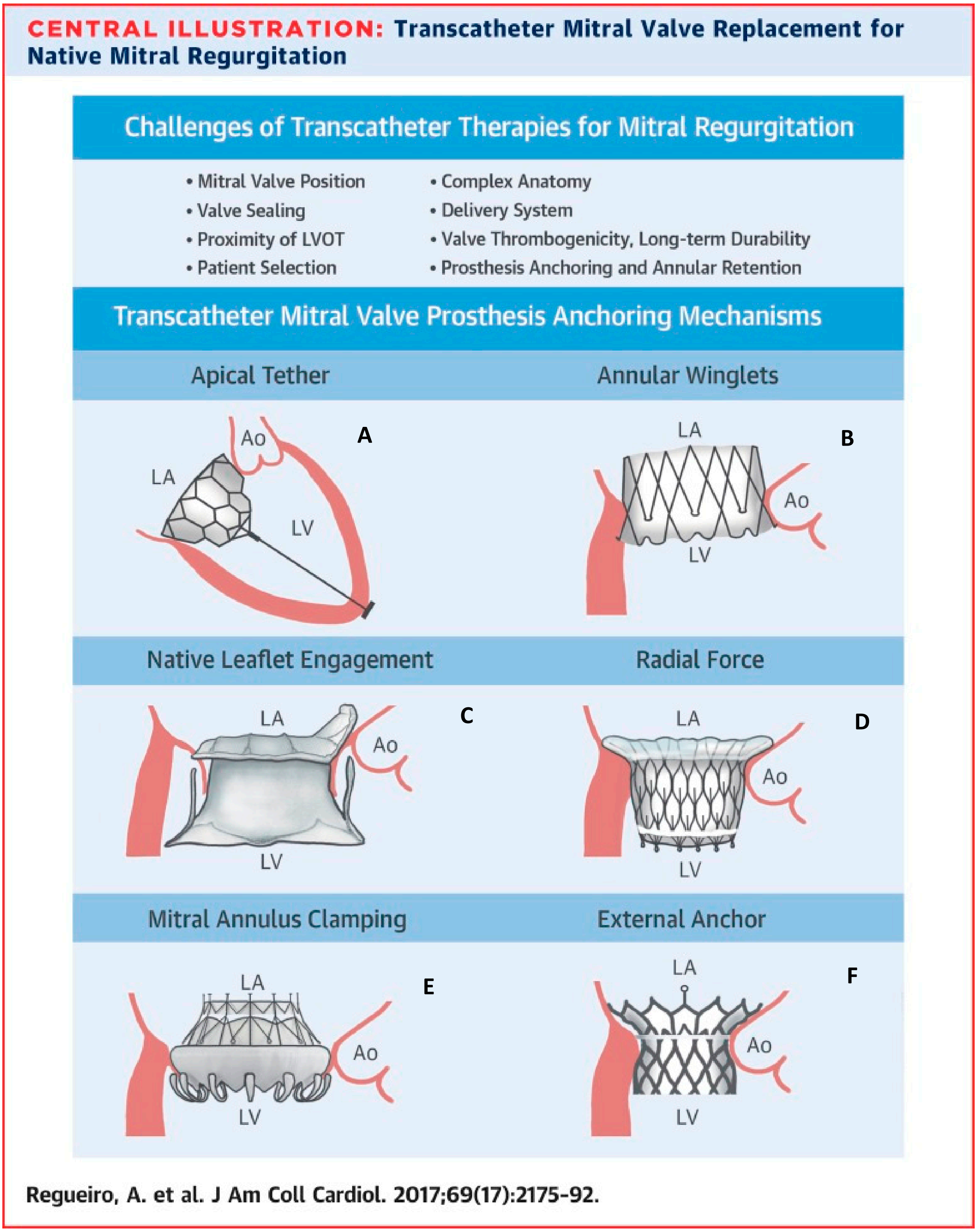


Figure S1. Transcatheter mitral valve anchoring mechanisms: apical tethering with an epicardial pad (A); atrial winglets or subannular piercing hooks (B); stent elements grasping native mitral leaflets (C); stent radial forces only (D); atrial and ventricular segments clamping leaflets/annulus (E); two-component systems with external docking elements (F). Reprinted from [1].

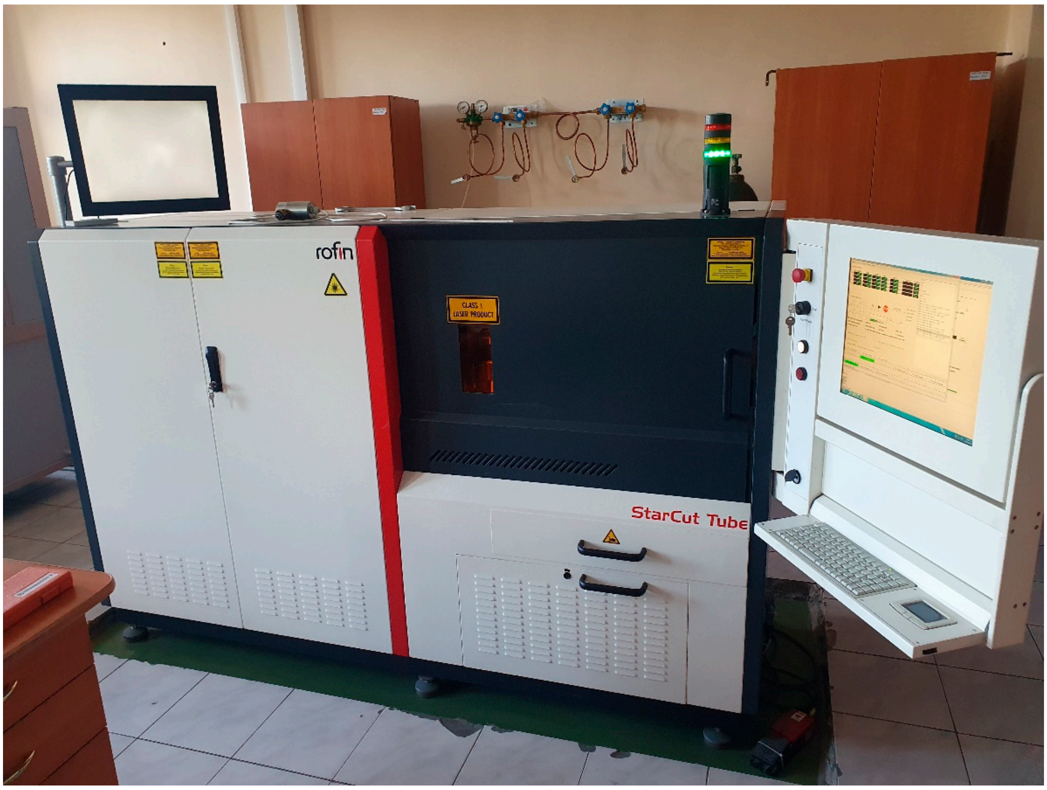


Figure S2. StarCut Tube femtosecond laser machine

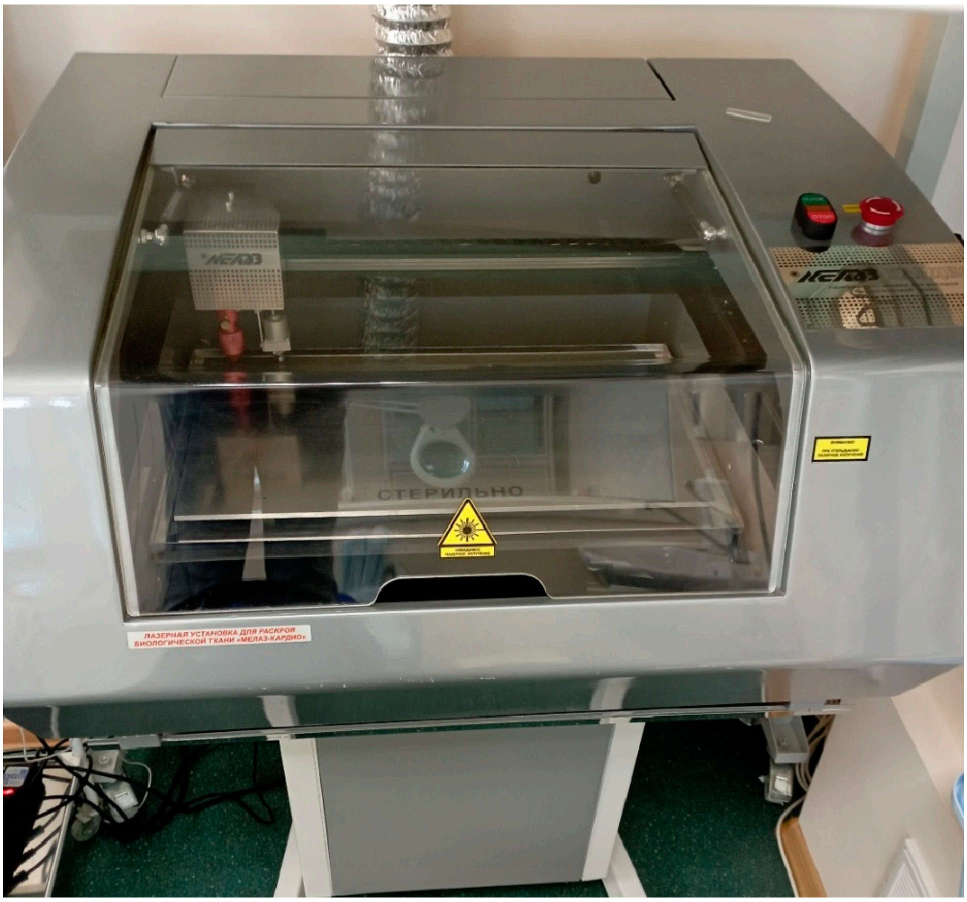


Figure S3. The biomaterial laser cutting machine “MELAS-Cardio”

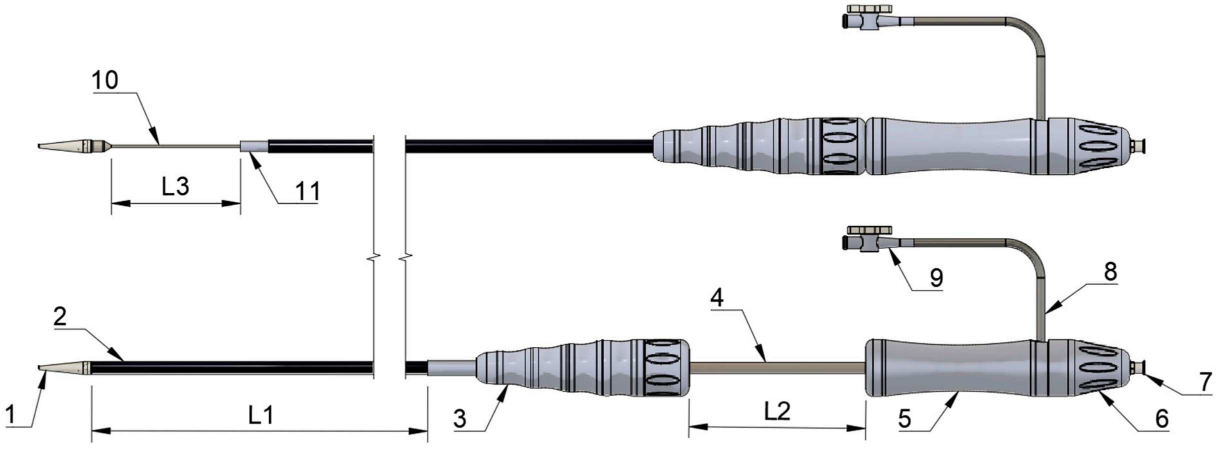


Figure S4. Transcatheter 22 Fr delivery system developed at the «Polytechnic» Science and Technology Park of the Belarusian National Technical University (Minsk, Belarus). Tapered tip 22 Fr (1), 22Fr sheath (L1 = 500 mm) (2), handle of the device (3 and 5), adjustment tube, working distance (L2=110 mm) (4), top of the handle (6), guide wire port (7), catheter tube (8), flush port (9), valve crimp section (L3=66 mm) (10), shaft (11).

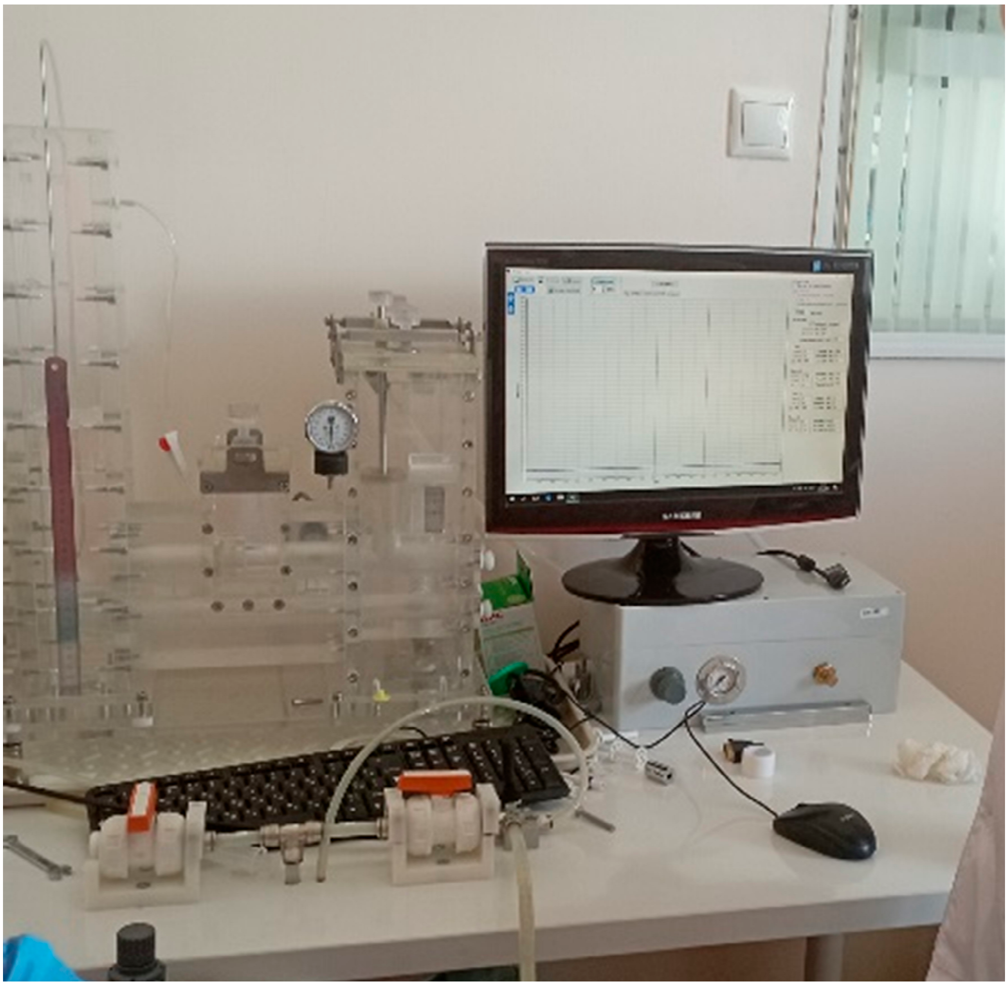

Figure S5. Pulsatile-flow tester

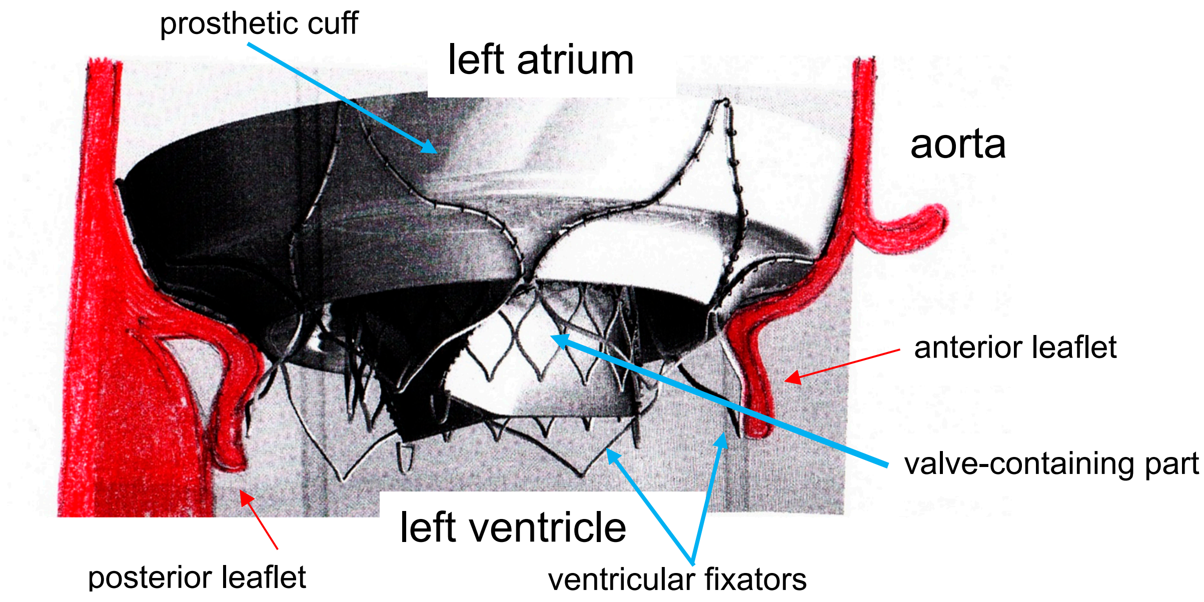


Figure S6. A schematic cross-section showing the anchoring zones and interface of the developed prototype with the mitral annulus, LV, and LA.

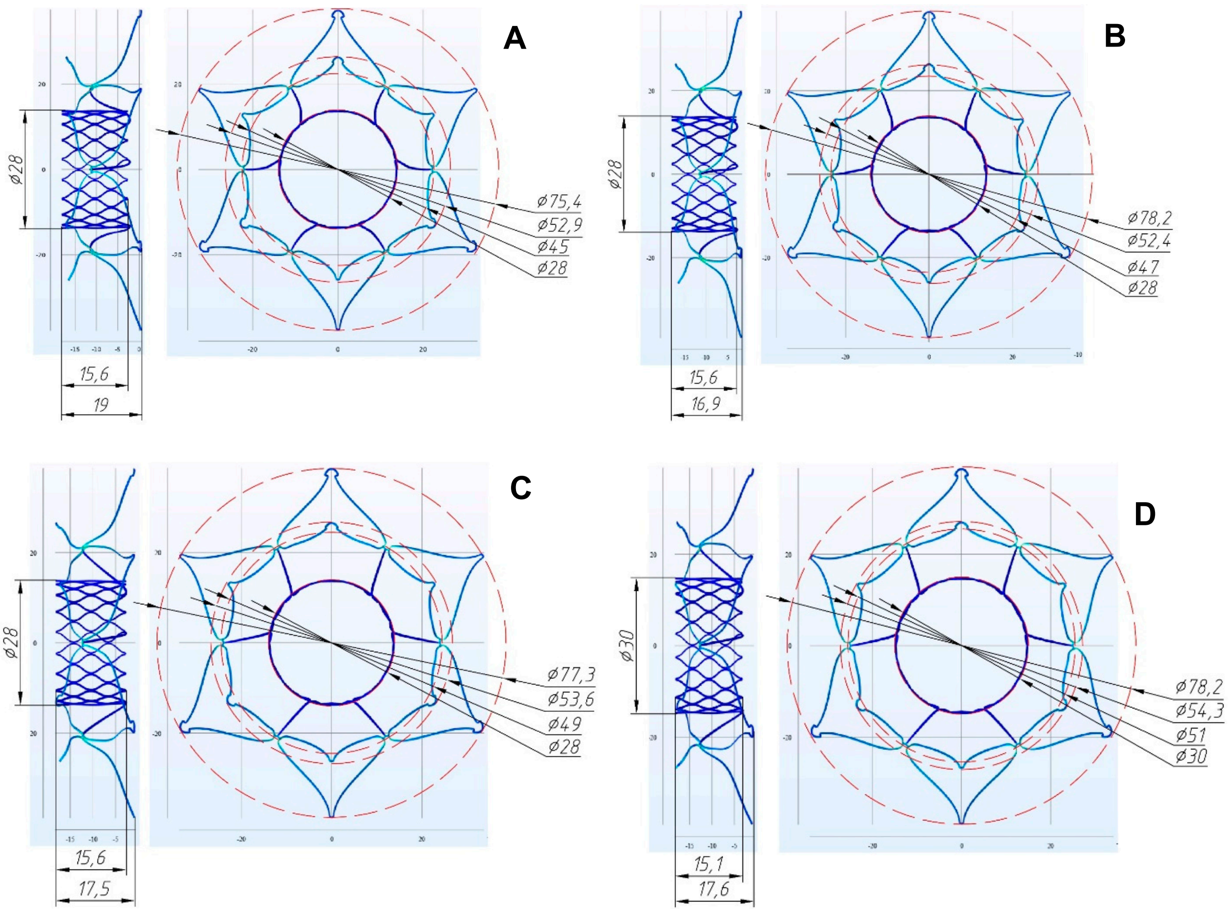


Figure S7. 3D models of stents for implantation in the mitral annuli of 40 mm (A), 42 mm (B), 44 mm (C), and 46 mm (D) in diameters.

## References

1. Regueiro, A.; Granada, J.F.; Dagenais, F.; Rodés-Cabau, J. Transcatheter Mitral Valve Replacement: Insights From Early Clinical Experience and Future Challenges. *J. Am. Coll. Cardiol*. **2017**, *69*, 2175–2192. https://doi.org/10.1016/j.jacc.2017.02.045.
